# Diabetes and technology – an update for the general physician

**DOI:** 10.1016/j.clinme.2025.100539

**Published:** 2025-12-05

**Authors:** Kyaw L.S. Khin, Alexandros L. Liarakos, Iskandar Idris, Ketan Dhatariya, Emma G. Wilmot

**Affiliations:** a Department of Diabetes and Endocrinology, University Hospitals of Derby and Burton NHS Foundation Trust, Royal Derby Hospital, Derby, UK; b School of Medicine, Faculty of Medicine and Health Sciences, University of Nottingham, Nottingham, UK; c Elsie Bertram Diabetes Centre, Norfolk and Norwich University Hospitals NHS Foundation Trust, Norwich; d University of East Anglia Medical School, Norwich, UK

**Keywords:** Diabetes, Technology, Inpatient care, Continuous glucose monitor (CGM), Insulin pumps, (Hybrid close loop) HCL, CSII

## Abstract

Diabetes is a growing public health concern. Approximately 20% of acute NHS hospital beds are occupied by individuals with diabetes. Following the recent NICE (National Institute for Health and Care Excellence) updates, diabetes technologies are increasingly available in the NHS. Despite the benefits, they present challenges, eg unfamiliarity, insufficient education, and lack of confidence of general physicians who are increasingly likely to encounter people using these technologies, presenting with acute illnesses.

This review aims to update the general physicians with diabetes technologies such as continuous glucose monitors, insulin pumps, hybrid closed loop systems and how to troubleshoot in acute illnesses, diabetes emergencies, perioperative management and radiological investigations.

While it is important to develop consistent inpatient care pathways and out-of-hours support from diabetes teams, it is vital to enhance the knowledge and confidence of non-diabetes physicians. Further research is warranted to support the use of technology in inpatient settings and diabetes emergencies.

## Graphical abstract

**Figure:**
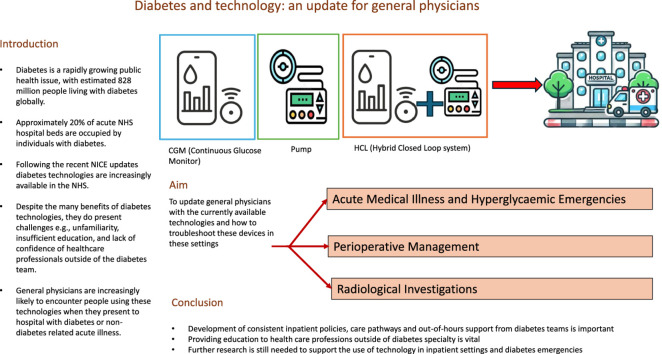

## Introduction

Diabetes mellitus is a global pandemic, growing rapidly in low-to-middle-income countries.^1^ An estimated 828 million people are living with diabetes worldwide, of whom 10% have type 1 diabetes (T1DM),^1^ with England reporting one of the highest rates of T1DM.^2^ Approximately one-fifth of acute hospital beds are occupied by individuals with diabetes.^2^

Diabetes technologies such as continuous glucose monitoring (CGM), continuous subcutaneous insulin infusions (‘insulin pumps’) and hybrid closed-loop (HCL) therapies have been shown to improve glycaemia and patient-reported outcomes in T1DM, with increasing evidence also suggesting benefits of these in type 2 diabetes (T2DM)^3^, ^4^, ^5^ .

Capillary blood glucose monitoring (CBG) monitoring was the standard of care before 2022, when NICE (National Institute for Health and Care Excellence) recommended CGM in all people living with T1DM and those with T2DM who are on multiple daily insulin injections with an elevated risk of hypoglycaemia, among other criteria.^6^^,^^7^ In 2024, NHS England published Technology Appraisal guidance on HCL use in T1DM, recommending HCL in 1) children and young people with T1DM, 2) those who are pregnant / planning pregnancy, 3) adults with T1DM with HbA1c ≥58 mmol/mol (7.5%) or disabling hypoglycaemia, despite best possible management with insulin pumps or CGM.^8^

These technological advances represent significant progress in diabetes care but also present challenges, including the need for healthcare professional education and upskilling, while for users there are possible issues related to alarm fatigue and anxiety.^9^ This article aims to familiarise general physicians with CGM, insulin pumps and HCL systems, and provide practical guidance for managing these devices in the inpatient setting until the diabetes team provides input.

## Continuous glucose monitoring (CGM)

**Fig. 1:**
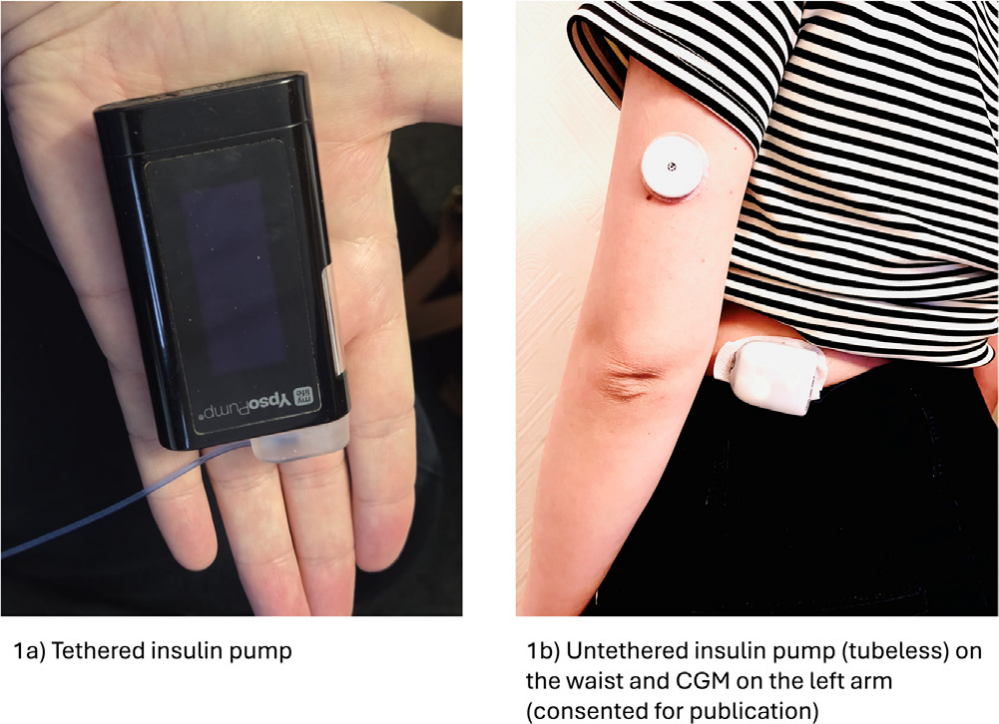
Examples of insulin pumps and continuous glucose monitoring sensor. 1a) Tethered insulin pump. 1b) Untethered insulin pump (tubeless) on the waist and CGM on the left arm (consented for publication). Image provided and published with the written consent of the individual depicted.

CGM sensors are coin-sized devices applied to the back of arm or abdomen. Real-time CGM (rtCGM) continuously transmits interstitial glucose data to a receiver or smartphone via Bluetooth.^10^ Each sensor typically lasts 7–15 days (depending on the manufacturer) and provides detailed data on glucose trends, enabling users to gain better insight into the impact of diet, lifestyle and therapy on their glucose levels, leading to improved self-management and behaviour modifications.^11^ CGM metrics such as time in range (TIR), time above range (TAR), time below range (TBR), glucose variability and glucose management index (GMI) are now routinely used in clinical care to guide diabetes management.^12^ CGM also provides the option of alarms for high and low glucose levels, particularly useful in those who have impaired awareness of hypoglycaemia symptoms. The different types of CGM available in current UK practice can be found in the diabetes specialist nurse (DSN) Forum UK platform.^13^ Fig. 1 demonstrates an example of an arm-worn CGM sensor.

## Continuous subcutaneous insulin infusions (CSII / insulin pumps)

An insulin pump consists of a reservoir, containing short-acting insulin, and a cannula which is typically worn on the abdomen, thigh or arm to deliver a continuous subcutaneous infusion of insulin.^10^ There are two types of insulin pumps: tethered insulin pumps, where a tube connects the pump and the cannula, and tubeless pumps, which connect directly to the skin as demonstrated in Fig. 1.^10^ Rapid-acting insulin is delivered continuously on an hourly rate (basal rate) throughout the day and after bolus activity by the user, with the dose based on insulin:carbohydrate ratio (ICR).^10^ Before initiation, individuals receive comprehensive education on insulin management, carbohydrate counting and troubleshooting.^14^ The DSN Forum UK platform describes the various types of insulin pumps used in the NHS.^13^

## Hybrid closed loop system (HCL)

Hybrid closed-loop (HCL) systems, also known as automated insulin delivery (AID) or artificial pancreas, deliver a variable rate of basal insulin via the insulin pump in response to rtCGM data, guided by mathematical algorithms to maintain target glucose levels.^10^ Bolus insulin administration with meals and snacks containing carbohydrates are still required with HCL systems. Omission of mealtime boluses can result in suboptimal glycaemic outcomes. In comparison to standalone CSII, HCL has been shown to improve HbA1c, reduce the risk of hypoglycaemia and reduce diabetes-related distress, leading to the NICE TA recommending wider access.^15^ Thus, NHS England is rolling this technology out to over 150,000 adults with T1DM in England as a part of their 5-year implementation plan.^16^

While CSII and HCL technologies are associated with improved glycaemic and patient-reported outcomes, their use comes with caveats. Insulin pumps deliver rapid-acting insulin without a long-acting insulin. This leads to a high risk of ketosis and progression to diabetes ketoacidosis (DKA) in the event of pump failure, tube blockage or cannula dislodgement. Hence, if unexpected hyperglycaemia occurs and infusion set or pump failure is suspected, people with diabetes are advised to promptly check ketone and glucose levels, followed by correction doses via subcutaneous insulin injection and change of their infusion set as per sick day rules.^14^

## Management of technology in acute medical illness and special situations

With the growing use of diabetes technologies in the diabetes population, general physicians are increasingly likely to encounter these devices in hospital settings. Below, we describe how a general physician should approach diabetes technologies during inpatient care, including the perioperative period and radiological investigations.

### Acute medical illness and hyperglycaemic emergencies

#### CGM

CGM can generally continue to be used in inpatients who are well enough to self-manage their device alongside CBG measurement.^14^ However, in those too unwell to self-manage their diabetes, CBG must be relied on.^14^ In patients who are haemodynamically unstable and/or experience hyperglycaemic emergencies (eg DKA), CBG or venous glucose measurements should be used for medical decisions,^14^^,^^19^ as currently there is not enough evidence to support the use of CGM data in diabetes emergencies.

In the inpatient setting, the blood glucose target should aim at 6–10 mmol/L (or 6–12 mmol/L) to reduce the risk of hypoglycaemia and treating for looming hypoglycaemia (4–6 mmol/L). Safety is important in the hospital; thereby setting high and low glucose alarms at 15–18 mmol/L and 4–5 mmol/L, respectively, will guide further CBG testing.^20^

Discrepancies can occur between CGM and CBG values, as CGM measures the interstitial glucose level as opposed to capillary blood. This may be more pronounced in the hospital setting due to factors like hypovolaemia, site infection, sensor placement, sensor wear time, calibration issues, extreme temperature, compression at the insertion site, peripheral oedema or physical activity, necessitating caution in inpatient use.^21^ Table 1 summarises scenarios warranting CBG verification.

**Table 1:** Inpatient situations to check capillary blood glucose while using a CGM device.^14^

Hypoglycaemia – to confirm hypoglycaemia and ensure it is treated
Symptoms not matching the sensor glucose level
Sensor errors – eg no reading or no arrow
Need for calibration
During and after exercise (physiotherapy sessions)
Acutely unwell
Hyperglycaemic emergencies
Incapacitated patients

#### Insulin pumps and hybrid closed loop

**Fig. 2:**
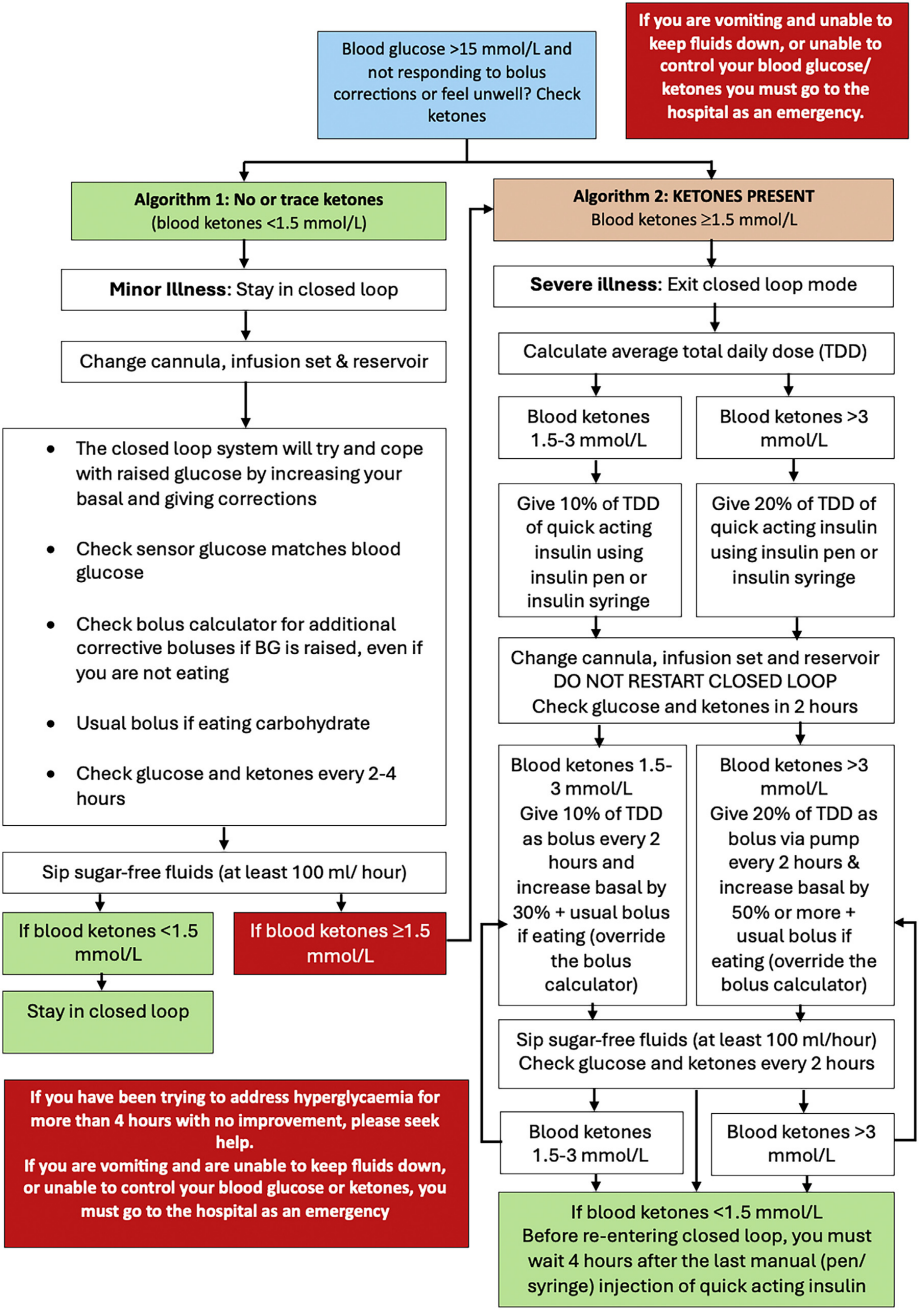
Unexplained hyperglycaemia and sick days rules (adapted with permission from ABCD DTN UK^15^^,^^17^^,^^18^).

**Fig. 3:**
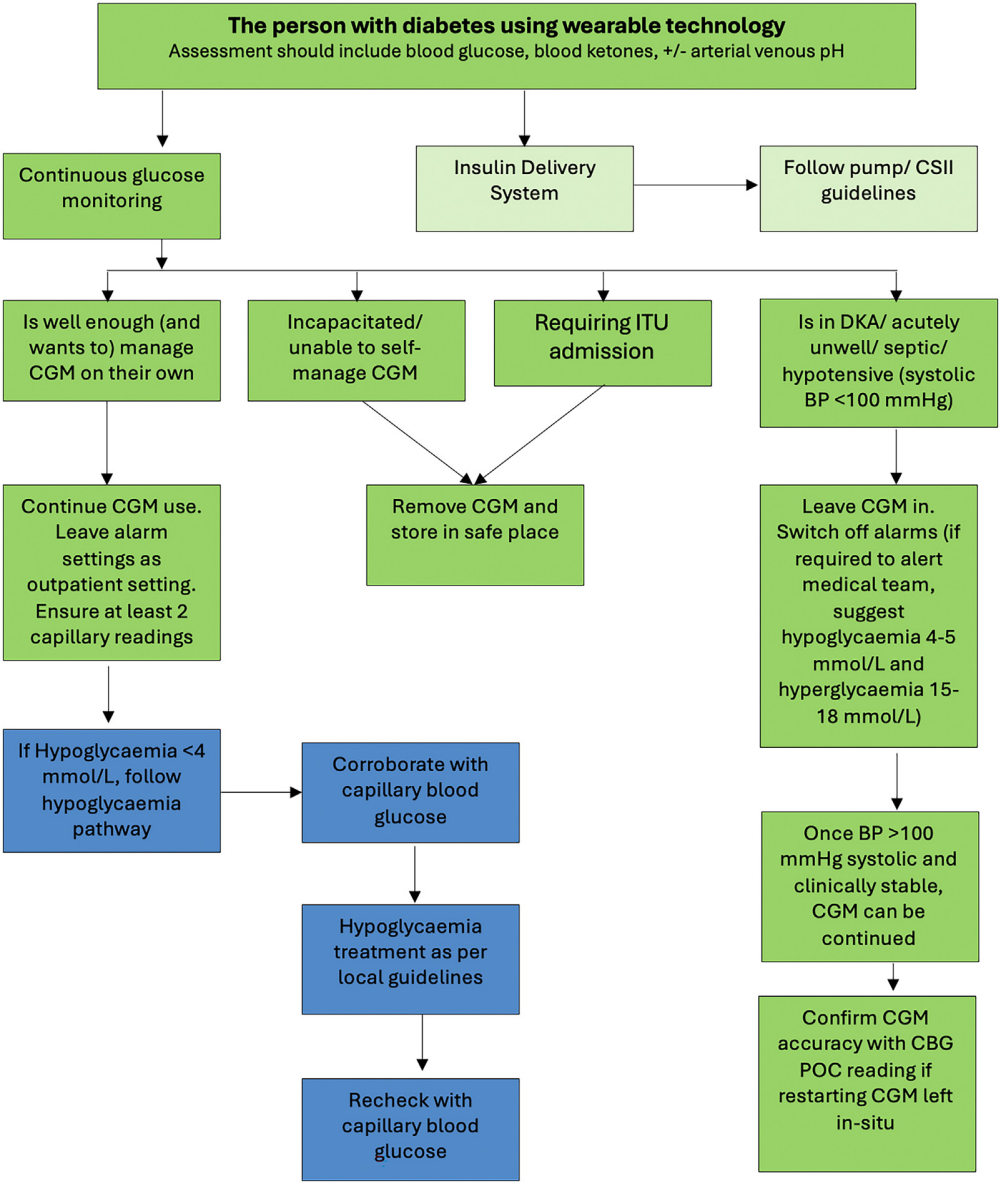
Recommendation for use of CGM in the hospital setting (adapted with permission from JBDS-IP guideline^14^).

**Fig. 4:**
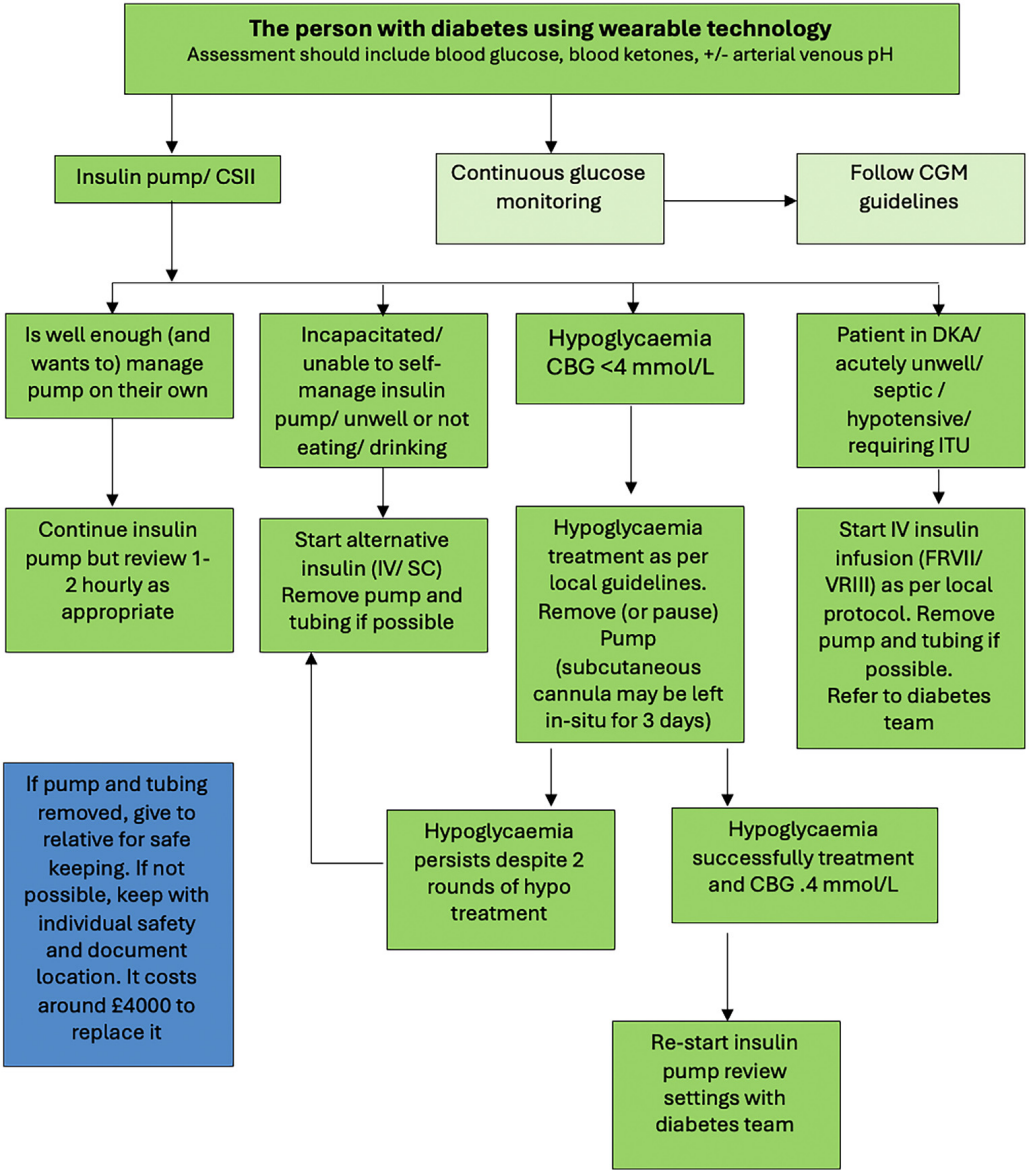
Algorithm for management of insulin pump in hospital setting (adapted with permission from JBDS-IP guideline^14^).

In critically unwell or incapacitated patients and during hyperglycaemic emergencies, insulin pumps should be removed, labelled and stored in a safe place. Intravenous (IV) insulin infusions should be initiated immediately, followed by a referral to the inpatient diabetes team at the earliest possible time.^14^ In stable patients who are able to manage their pumps, use of CSII may be continued under close supervision.^14^ For HCL systems, if patients are unwell with ketosis, the system should be switched from automated to manual mode (which stops communication between CGM and pump, working as standalone pump and sick day rules should be followed) (Fig. 2, Fig. 3, Fig. 4).^14^

When switching from CSII to insulin infusion or insulin injections, it is important to administer the alternative form of insulin as soon as possible (within 1 hour of stopping CSII).^22^ On restarting CSII, there should be a 60-minute overlap between IV insulin and CSII ensuring that glucose and ketones are not rising.^22^ Patients should be reminded to give correction insulin if CBG >10 mmol/L using their usual insulin sensitivity factor.^22^ If CSII is to be restarted after subcutaneous basal insulin, the basal rate of CSII should be reduced to 70% for the first 12–24 h until the effect of long-acting insulin wears off.^22^ More frequent monitoring is required during the transition period, starting with 1–2 hourly initially until target range is achieved.^22^

### Perioperative management

#### CGM

CGM can be continued during minor procedures (eg endoscopies). In major procedures (>1 missed meal), CGM should be placed away from the operative site and diathermy and may be used to guide blood glucose monitoring frequency, but should not be used for treatment decisions. Blood glucose readings should guide treatment decisions. CGM should be avoided in cases of intraoperative hypotension or haemorrhage.^14^

#### Insulin pumps (CSII)

Insulin pumps may continue during minor procedures with standard basal rates. Devices should be kept away from diathermy sites, using Teflon cannulas only.^14^ For major surgeries, insulin pumps should be discontinued and stored in a safe place, and variable rate insulin infusion (VRII) should be administered.^14^

### Radiological investigations

#### CGM

CGM may remain in place during X-ray and CT scans if adequately shielded, depending on local radiology department guidance.^14^ However, CGM should be removed for MRI procedures due to the risk of device displacement.^14^

#### Insulin pumps (CSII)

Insulin pumps may also be continued during X-ray and shielded during CT scans. For MRI, the insulin pump should be removed.^14^

## Conclusion and future directions

The use of diabetes technologies is increasingly widespread, with general physicians encountering more patients using these devices. However, the development of inpatient care pathways and out-of-hours support from diabetes teams remains inconsistent across hospitals.^9^^,^^20^ Additionally, other diabetes technologies such as smart connected pens, digital platforms and the use of virtual reality living are also expanding rapidly. Hence, it is essential for HCPs to broaden their understanding of these technologies. Providing education to HCPs outside the diabetes specialty is vital to support patients using such devices. Improving access to CGM reports via electronic health records may enable general physicians to make insulin adjustments during inpatient care. Nonetheless, further research is needed to support the use of technology in inpatient settings and diabetes emergencies. Such advancements could help reduce the burden to HCPs and improve the care for people living with diabetes by reducing the amount of CBG testing and optimising insulin dosing.

## Funding

This research did not receive any specific grant from funding agencies in the public, commercial, or not-for-profit sectors.

## Consent for publication

Written informed consent was obtained from the individual depicted in Fig. 1.

## CRediT authorship contribution statement

**Kyaw L.S. Khin:** Writing – review & editing, Writing – original draft, Visualization, Conceptualization. **Alexandros L. Liarakos:** Writing – original draft, Conceptualization. **Iskandar Idris:** Writing – review & editing. **Ketan Dhatariya:** Writing – review & editing. **Emma G. Wilmot:** Writing – review & editing, Supervision, Conceptualization.

## Declaration of competing interest

The authors declare the following financial interests/personal relationships which may be considered as potential competing interests: Emma G Wilmot reports a relationship with Abbott, Embecta, Insulet, Novo Nordisk, Sanofi, AstraZeneca, Dexcom, Eli Lilly, Medtronic, Roche, Sanofi, Sinocare, Ypsomed, Tandem that includes: consulting or advisory, funding grants, speaking and lecture fees. Ketan Dhatariya reports a relationship with AstraZeneca, Böhringer, Ingelheim, Eli Lilly, Abbott Diabetes, Menarini, Sanofi Diabetes, Roche that includes: consulting or advisory, speaking and lecture fees, and travel reimbursement. Iskandar Idris reports a relationship with Abbott, Boehringer, Eli Lilly, Novo Nordisk, Astra Zenica, Sanofi that includes: consulting or advisory, funding grants, and speaking and lecture fees. Alexandros L Liarakos reports a relationship with Dexcom, Novo Nordisk, ABCD that includes: funding grants, speaking and lecture fees, and travel reimbursement. If there are other authors, they declare that they have no known competing financial interests or personal relationships that could have appeared to influence the work reported in this paper.
